# Anterior Cruciate Ligament Reconstruction Rehabilitation as a Complex Adaptive Process: From Control–Chaos to Actionable Return-to-Sport Decisions

**DOI:** 10.3390/bioengineering12111229

**Published:** 2025-11-10

**Authors:** Georgios Kakavas, Nikoloaos Malliaropoulos, Florian Forelli

**Affiliations:** 1 Fysiotek Spine & Sports Lab, 17562 Athens, Greece; georgios.kakavas@gmail.com; 2 Thessaloniki Sports Medicine Clinic, 54622 Thessaloniki, Greece; 3 Sports Clinic, Rheumatology Department, Barts Health NHS Trust, London E1 1BB, UK; 4 Haute-Ecole Arc Santé, HES-SO University of Applied Sciences and Arts Western Switzerland, 2800 Delémont, Switzerland; 5 Orthopaedic Surgery Department, Clinic of Domont, Ramsay Healthcare, OrthoLab, 95330 Domont, France; 6 Société Française des Masseurs—Kinésithérapeutes du Sport Lab, 93380 Pierrefite sur Seine, France

**Keywords:** anterior cruciate ligament reconstruction, adaptive periodization, complexity, chaos

## Abstract

Rehabilitation after anterior cruciate ligament reconstruction cannot be reduced to a linear, time-based sequence of protection, strength, and return to sport. Persistent asymmetries, quadriceps inhibition, and variable re-injury rates highlight that recovery is a complex adaptive process in which outcomes emerge from dynamic interactions between biological, neural, and psychological subsystems. Grounded in complexity science and chaos theory, this editorial reframes rehabilitation as the regulation of variability rather than its suppression. The Control–Chaos Continuum provides a practical structure to translate this concept into progressive exposure, where clinicians dose uncertainty as a therapeutic stimulus. Adaptive periodization replaces rigid stages with overlapping macro-blocks that respond to readiness, feedback, and context. Neuroplastic mechanisms and ecological dynamics justify the deliberate introduction of controlled “noise” to foster coordination, confidence, and resilience. Ultimately, the goal is not perfect control but stable performance under variability—the ability to function “at the edge of chaos.” This conceptual perspective articulates a clinically actionable framework—linking the Control–Chaos Continuum with adaptive periodization—to guide non-linear decision-making and safe return-to-sport.

## 1. Introduction—Complexity and the Illusion of Linear Recovery

Rehabilitation after anterior cruciate ligament reconstruction (ACLR) is often presented as a tidy sequence of protection, strength, function, and return to sport. This representation gives clinicians and athletes a sense of certainty, yet real recovery rarely conforms to such linear logic. Persistent quadriceps inhibition, lingering asymmetries, and variable re-injury rates across cohorts show that standardized protocols can produce very different trajectories in practice [1,2,3,4]. In the context of ACLR rehabilitation, the athlete functions as a complex adaptive system—a continually reorganizing network of neuromuscular, sensory, psychological, and mechanical subsystems—such that divergences are not noise but evidence that recovery emerges from their interactions rather than from any single intervention [5].

In a complex system, small differences in initial conditions can produce large, path-dependent consequences. A slight shift in pain sensitivity, sleep, motivation, or load tolerance can reorganize the way a person moves, learns, and regains confidence. Linear cause-and-effect thinking struggles to capture this reality because it underestimates feedback, context, and time-dependent learning [6]. Complexity thinking, in contrast, treats variability as information and focuses on how constraints shape behavior. Physiotherapy therefore transitions from delivering a protocol to designing conditions under which the system can self-organize toward stability and performance [7,8,9].

Chaos theory provides the conceptual bridge. In deterministic chaos, systems follow rules yet display unpredictable trajectories because they are exquisitely sensitive to initial conditions. Two athletes with similar surgery, graft, and program can diverge entirely due to small differences in neuromuscular inhibition, attention, or perceived threat. The clinician’s task is not to eliminate variability but to regulate it so learning occurs without collapse. Optimal adaptation tends to occur at the edge of chaos (functional boundary where the system operates between stability and instability. It is the point at which the task is challenging enough to stimulate adaptation and learning, yet not so unpredictable that coordination collapses or symptoms emerge), a region where there is enough order for safety and enough disorder for exploration and neuroplastic change [10,11,12].

Recognizing recovery after ACLR as complex and adaptive reframes clinical expertise. The therapist becomes a regulator of constraints—modulating task, environment, and feedback—so that the individual system can discover robust solutions under changing conditions. Readiness is no longer defined by calendar time alone but by the capacity to maintain function while variability increases in a controlled manner [1,2,3].

This conceptual perspective synthesizes prior models from complexity science, ecological dynamics, and sports rehabilitation to advance a unified clinical framework for ACLR. Our objective is to (i) define a common language for clinicians around control, variability, entropy, and the “edge of chaos”; (ii) operationalize the Control–Chaos Continuum through adaptive periodization and task representativeness; and (iii) provide practice-oriented principles that support non-linear decision-making and safer return-to-sport pathways.

## 2. Why Control–Chaos Continuum Matters in ACLR Rehabilitation

The Control–Chaos Continuum articulated by Taberner et al. translates complexity into practical progression. Early after ACLR, therapy emphasizes control: predictable movements, slow tempos, stable bases of support, and clear feedback that re-establish safe coordination. As tolerance grows, the environment is deliberately made less predictable with graded perturbations, dual-task demands, time pressure, and representative drills that more closely resemble sport [13,14]. The aim is not to provoke failure but to test whether coordination survives under stress and rapidly restabilizes. Variability is treated as a therapeutic dose rather than as an undesirable fluctuation [15].

Performance oscillations within this process are expected. A step backward after a new perturbation does not necessarily indicate regression. Instead, it often marks the system’s exploration of its limits and its search for more efficient solutions. The meaningful marker of readiness is the capacity to recover stability after disturbance, not perfect execution in simplified conditions [15,16]. This criterion aligns with how robust behavior is defined in complex systems: not by the absence of error, but by the ability to tolerate and correct it.

The word “chaos” suggests disorder, but biological chaos is structured unpredictability. Healthy movement exhibits fractal fluctuations across time—variability that is neither random nor rigid. Too little variability narrows the solution space and invites overload; too much undermines efficiency and control. Effective rehabilitation aims for an optimal bandwidth of variability that is wide enough to stimulate adaptation yet bounded enough to ensure safety [16,17,18]. In practice, this means intentionally manipulating information, constraints, and context to steer behavior rather than prescribing singular “ideal” techniques.

This concept matches ecological dynamics, which holds that movement emerges from the coupling between the individual, the task, and the environment. Changing any element—surface properties, rules of a drill, timing windows, or attentional cues—alters the available affordances and invites new coordinative solutions [1,19]. Neuroscience further supports controlled variability. Small amounts of sensory noise can enhance signal detection and learning through stochastic resonance; coupled with variable practice and reactive decision-making, such noise appears to accelerate neuroplastic reweighting of sensory–motor loops [20,21]. In other words, well-dosed unpredictability is a feature, not a bug, of effective learning after ACLR.

## 3. From Fixed Stages to Adaptive Periodization

Traditional rehabilitation borrows from classical periodization in sport, organizing work into predictable cycles of load and recovery. After ACLR, however, adaptive capacity fluctuates with pain, inhibition, fatigue, sleep, and emotion. A calendar that dictates when to progress can easily misalign with what the system can tolerate that day. Athletes who are resilient may be under-challenged; those who are vulnerable may be overexposed [22,23].

An adaptive periodization model reframes progression as readiness-based rather than time-based. Instead of rigid stages, the process unfolds through overlapping macro-blocks defined by functional goals—activation, strength, control, chaos, and reintegration. Advancement does not depend on weeks post-surgery but on observed adaptability across multiple indicators. When a person absorbs a new dose of variability without losing coordination or escalating symptoms, challenge expands. When instability dominates, intensity regresses to restore control [24].

Campardo et al. formalized how these macro-blocks interlock. Neuromuscular activation may still be consolidating during strength work; psychological readiness may advance even as mechanical tolerance lags; control and chaos co-exist in the same week but in different sessions or contexts. Macro-blocks are therefore temporary attractor states, not calendar compartments. They overlap and interact because the biological, neural, and psychological subsystems adapt asynchronously [25,26,27].

Clinicians function as architects of constraints. They adjust task difficulty, velocity windows, fatigue, and informational complexity to ensure variability remains productive. Introducing complex perturbation too early can destabilize; maintaining rigidity too long suppresses exploration and delays reintegration. Adaptive periodization explicitly connects load management with neural recovery: classic programming alternates stress and rest to optimize tissue adaptation, whereas adaptive programming alternates control and chaos to optimize system adaptability [28,29]. Evidence from tendinopathy, hamstring rehabilitation, and neurorehabilitation suggests that flexible, feedback-driven dosing outperforms rigid prescriptions when variability and readiness are respected [30,31,32].

This perspective also incorporates social and cognitive factors. Confidence, perceived threat, attention, and motivation modulate motor output as strongly as mechanical loading. By embedding challenge in meaningful tasks—game-like constraints, time pressure, competitive elements—clinicians stimulate the same circuits that support decision-making, anticipation, and self-efficacy. The athlete learns not only to move but to trust the movement in uncertain environments [33].

## 4. Neuroplasticity and Variability as Drivers of Recovery

Anterior cruciate ligament injury does not merely disrupt a passive restraint; it alters afferent signaling and reshapes cortical representations involved in proprioception and motor planning. Neuroimaging demonstrates persistent asymmetries in cortical excitability and network connectivity long after surgical healing. These central adaptations influence timing, muscle recruitment, and perception of threat during movement [34,35,36,37]. Rehabilitation must therefore treat the nervous system, not just the knee. It needs varied sensory information, error signals, and contextual richness to recalibrate predictive control [20,38].

Clinical studies repeatedly show that voluntary activation and coordination can remain abnormal even when strength appears symmetrical. Moiroux-Sahraoui et al. documented asynchronous quadriceps patterns during single-leg tasks after ACLR, while Forelli et al. emphasized that arthrogenic muscle inhibition persists well beyond early recovery and can blunt performance unless directly addressed. Surface electromyography feedback, perturbation training, and dual-task exposure inject “useful noise” that helps the cortex and spinal circuits re-establish efficient communication [39,40,41]. Rather than treating variability as a failure to control, these methods use variability to retrain control.

Non-elite football players assessed months after clearance still show asymmetries in strength, balance, and movement quality. Such findings confirm that readiness cannot be inferred from symmetry ratios or hop distances alone. The decisive test is the ability to maintain coordination under changing conditions: adaptive stability in the face of uncertainty [42]. This criterion matches the Control–Chaos Continuum and the logic of adaptive periodization, which both require proof of stability while information and task variability increase [13,24].

## 5. Operationalizing the Control–Chaos Continuum

### 5.1. Closed-Loop Assessment: From Isolated Signals to Converging Evidence

**Figure 1:**
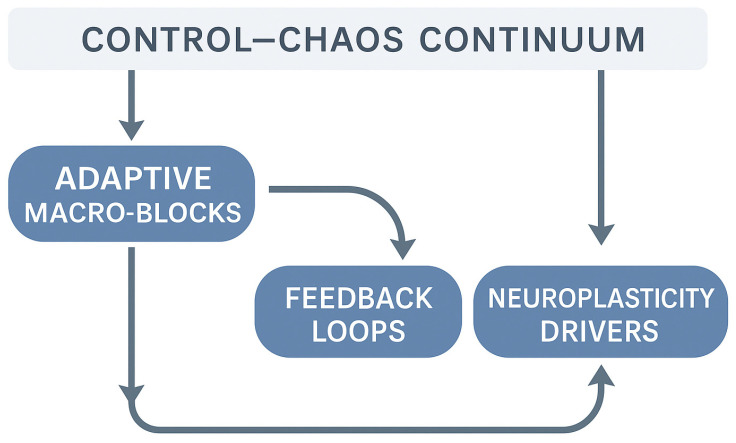
Operational framework linking the Control–Chaos Continuum to adaptive rehabilitation processes.

Operationalizing the continuum begins with a genuinely multimodal assessment, conceived as a closed-loop process that links measurement, interpretation, and clinical decision-making (Figure 1). Rather than relying on a single “perfect” metric, clinicians evaluate the convergence of mechanical indicators (peak torque, rate of force development), neuromuscular and coordination signals (surface Electromyography patterns, co-activation, temporal stability of motor signatures), behavioral markers (decision speed, error dispersion), load tendencies (acute-to-chronic ratios), and psychological disposition (confidence, perceived threat). Taken together, these elements reveal the athlete’s true adaptive stability, meaning their capacity to maintain coordination as informational demands and uncertainty increase. Within this framework, surface Electromyography and coordination-sensitive tasks act as early sentinels: they often expose control deficits that remain hidden when strength appears symmetrical, and they guide the type of exposure required to reconnect voluntary activation, movement quality, and tolerance to variability [40,43].

### 5.2. Dosing Complexity: Manipulating Constraints to Regulate Entropy

The exposure component consists of systematically manipulating task and environmental constraints (planes of movement, speed windows, external cues, density of visual information, opponent interactions, time pressure) to modulate functional entropy (level of task variability and unpredictability within the rehabilitation environment. Low-entropy tasks are stable, predictable, and closed; high-entropy tasks contain uncertainty, multi-directional demands, or reactive decisions, challenging the athlete’s ability to maintain coordination). Early sessions prioritize controllability, focusing on quadriceps reactivation, secure motor patterns, and strong sensorimotor anchors within predictable tasks. Once stability is consistently observed, clinicians introduce structured variability (graded multidirectionality, controlled perturbations, variable speed windows) to strengthen the robustness of the athlete’s movement solutions. The next stage is contextual chaos, defined by reactive decision-making, spatial–temporal unpredictability, and real-time coupling with the environment. Finally, sport-specific chaos reproduces the affordances of the target sport (e.g., pressing schemes, visual scanning demands, aerial duels), enabling clinicians to evaluate whether performance remains stable under the same uncertainties encountered in competition [44,45]. At each stage, the guiding criterion is not the absence of error, but the athlete’s capacity to re-stabilize coordination quickly after perturbation.

### 5.3. Inflection Points and Recalibration: Advancing When Errors Become Information

Recalibration occurs immediately based on the athlete’s responses. If technical quality, error dispersion, and symptom provocation remain within acceptable ranges, the clinician expands the dose of uncertainty. If coordination becomes fragile, load and complexity are rolled back to restore a stable attractor. Practically, an inflection point is reached when a new increment in variability does not destabilize kinematics, electromyography signatures, or behavioral regularity beyond a predetermined corridor. This logic ensures that progression remains reversible and safe, allowing the athlete to operate near the edge of chaos—a zone containing enough order for safety and enough disorder for plasticity and learning [14,24,46].

### 5.4. Task Representativeness and Chaotic Load

The predictive power of linear or “clean” tests remains limited when they fail to capture adaptability under temporal and informational constraints. Operationalizing the continuum requires representative tasks, in which success depends on meaningful perception–action coupling: unplanned changes of direction, unpredictable visual or auditory cues, space-sharing with an opponent, object handling, and narrow decision-making windows. Within these contexts, three families of indicators quantify chaotic load: deceleration and horizontal braking capacity, rapid force production, and asymmetry indices in movement and activation patterns. Monitoring these clusters reveals the robustness of the athlete’s motor solution beyond maximal strength alone and guides the granularity of complexity increments from one session to the next [44,45].

### 5.5. Integration with External/Internal Load and the Psychological Dimension

The continuum’s macro-blocks overlap: athletes can continue developing strength while increasing exposure to chaos, and psychological readiness may improve through successful representative experiences even as mechanical tolerance is still being protected. Acute-to-chronic ratios help differentiate functional adaptation from accumulating stress; they do not replace clinical judgment grounded in converging signals, but they contribute to securing the trajectory. Meanwhile, psychological readiness develops through graded mastery experiences that desensitize threat, transform uncertainty into usable information, and strengthen agency. It is this coordinated orchestration of load, coordination quality, and confidence that allows the system to learn to tolerate variability without collapsing [47,48,49].

### 5.6. Biological Rationale: Directed Plasticity and Reorganization

From a mechanistic perspective, ACLR disrupts central integration, and controlled exposure to variability provides the prediction errors and sensory richness required to re-weight sensorimotor loops. Adaptive periodization makes this plasticity clinically actionable by linking each increment in entropy to observable indicators and embedding reversible steps when stability deteriorates. The objective is not perfect execution in simplified tasks, but stable performance under variability—the competence to operate reliably near the edge of chaos. This criterion bridges theory and field-based decisions and justifies evaluating an athlete’s capacity to maintain and recover coordination rather than relying on calendar-based milestones or isolated symmetry ratios [34,35,40].

### 5.7. Ethical and Educational Implications

During phases that involve greater variability or “chaos exposure,” transparent communication between clinician and patient becomes essential. We emphasize the need to clearly explain the purpose of these high-uncertainty tasks, the expected sensations, and the protective constraints in place. Discussions around risk tolerance, perceived safety, and progression boundaries help ensure that the patient remains an active agent in the process rather than a passive recipient. In this context, shared decision-making and informed consent are central, allowing the patient to understand how controlled unpredictability contributes to learning, confidence, and functional robustness.

## 6. Future Directions

Although this manuscript is primarily conceptual, several empirical observations support the relevance of the Control–Chaos Continuum in ACLR rehabilitation. Clinically, persistent neuromuscular asymmetries, altered coordination patterns, and increased variability under reactive or unplanned conditions are consistently observed months after ACLR and justify progressive exposure to complexity. Emerging evidence on deceleration capacity, horizontal braking, and reactive change-of-direction behaviours also indicates that athletes with better adaptability under variable constraints demonstrate more robust functional performance. In applied settings, physiotherapists frequently report improvements in movement quality, confidence, and transfer to sport tasks when progressions are guided by graded complexity rather than linear stage-based rehabilitation. These practice-based insights provide preliminary support for the continuum as a clinically meaningful approach.

ACLR rehabilitation is an ideal arena for translational complexity, where systems science meets the messy reality of clinical practice. Future research should map how kinematic entropy, surface electromyography regularity, decision-time variability, and workload trends co-evolve during recovery. Longitudinal, real-world datasets would allow non-linear modeling of adaptability and help identify tipping points between productive challenge and overload [25,40,50]. Such models could move decision-making beyond thresholds and toward pattern recognition across domains.

Digital tools make this feasible. Wearable sensors, instrumented fields, and cloud analytics can capture movement and neural signatures in ecological contexts. Real-time dashboards could support adaptive dosing of variability by flagging when coordination is robust to perturbation or when error dispersion widens unsafely. The concept of a digital twin—a computational avatar that predicts how a person might respond to specific constraint changes—could soon assist in planning progressions and avoiding plateaus or spikes in risk [51,52].

Complexity reframes uncertainty as useful information rather than error. Systems that show modest fluctuations are often more adaptable than those that appear perfectly stable, because they retain the flexibility to reorganize. Clinicians should therefore learn to read variability instead of suppressing it, cultivating a tolerance for ambiguity that is disciplined by measurement and guided by safety. In this paradigm, the therapist acts as a navigator of instability, steering the athlete through a safe corridor of chaos where learning accelerates without collapse [53,54].

Embedding this mindset in practice requires cultural change. Physiotherapy curricula should teach systems thinking, data literacy, and adaptive reasoning so new clinicians can manage feedback loops rather than implement fixed recipes. Educational frameworks can emphasize representative learning design, decision-making under time pressure, and interpretation of variability across strength, control, and confidence. Authors such as Ardern and Welling argue for readiness as multidimensional convergence rather than a single cut-off, a stance that aligns with complexity-informed practice [43,53]. Incorporating these principles into continuing education and team workflows would help align day-to-day practice with the science of adaptation.

Working at the edge of chaos blends scientific precision with clinical artistry. Objective measures quantify where the system stands; empathy, timing, and context help decide what to do next. Forelli et al. highlight that return-to-sport decisions should rest on coherent patterns across domains rather than on isolated metrics. Mastery consists of controlling chaos without extinguishing it—designing environments where variability teaches stability and where the athlete’s confidence returns because coordination proves resilient in authentic conditions [40].

The value of complexity-informed rehabilitation extends beyond ACLR. The same logic—measure feedback, manipulate constraints, and cultivate adaptive variability—applies to many musculoskeletal and neurological conditions. By embracing uncertainty as data and variability as a tool, physiotherapy can evolve into a science of guided self-organization. Such a perspective does not discard protocols; it situates them within a flexible framework that respects how living systems actually change.

## 7. Take-Home Messages

Rehabilitation after ACLR is a complex adaptive process, not a linear checklist. The Control–Chaos Continuum and adaptive periodization make variability actionable, transforming it from a threat into a driver of neuroplasticity and resilience. Readiness should be inferred from multidimensional feedback and the capacity to maintain performance under graded unpredictability, not from calendar time or symmetry alone. Clinicians act as regulators of chaos, using constraints and feedback to help athletes thrive where control and uncertainty meet [1,4,7,8,9,10,11,12,13,14,20,24,25,26,27,28,34,35,36,40,44,45,46,47,48,49,50,51,52,53,54,55].

## Data Availability

No new data were created or analyzed in this study.
